# Effect of genotype of growing rabbits on productive performance with special reference to residual feed intake at hot temperature

**DOI:** 10.5713/ab.22.0355

**Published:** 2023-02-27

**Authors:** Moataz Fathi, Magdy Abdelsalam, Ibrahim Al-Homidan, Osama Abou-Emera, Gamal Rayan

**Affiliations:** 1 Department of Animal Production and Breeding, College of Agriculture and Veterinary Medicine, Qassim University, Buraydah, Al-Qassim 51452, Saudi Arabia; 2 Department of Poultry Production, Faculty of Agriculture, Ain Shams University, Hadayek Shoubra 11241, Cairo, Egypt; 3 Department of Animal and Fish Production, Faculty of Agriculture, Alexandria University, El-Shatby 21545, Alexandria, Egypt; 4 Agriculture Research Centre, Animal Production Research Institute, Dokki, Giza 12618, Egypt; 5 Department of Animal and Fish Production, College of Agricultural and Food Sciences, King Faisal University, Al-Ahsa 31982, Saudi Arabia

**Keywords:** Correlation Coefficient, Hot Climate, Rabbit Breed, Regression Analysis, Residual Feed Intake

## Abstract

**Objective:**

Better feed efficiency can be achieved by selecting rabbit genotypes with lower residual feed intake (RFI) under high ambient temperatures.

**Methods:**

Two genotypes of rabbits (Jabali, Saudi local breed and imported, Spanish V-line) were used to derive RFI and to investigate the relationship between RFI and productive traits. In total, 250 animals (125 each) were housed in individual wire mesh cages in a semi-closed rabbitry. Growth performance, feed criteria, carcass evaluation, biochemical blood analysis, and immune responses were determined.

**Results:**

Superiority in growth performance, feed efficiency, carcass characteristics, and cellular immunity was recorded in the Jabali breed compared to the V-line genotype. According to regression analysis, a significant effect of daily body weight gain was found, upon computing the expected feed intake in both genotypes. Moreover, mid-body weight^0.75^ had a significant effect only in the Jabali breed. Positive correlation coefficients between RFI and dry matter feed intake or feed conversion ratio were found. The same trend in this relationship between RFI and productive traits was observed in some cases for both genotypes. An opposite trend in correlations was observed in the studied genotypes for some traits.

**Conclusion:**

The results suggest that the relationship between RFI and productive traits must be taken into consideration in rabbit breeding programs under the prevailing environment. However, further studies are required to investigate the effect of rabbit genotype and environmental factors on computing RFI.

## INTRODUCTION

There is no doubt that the performance of growth and productivity in rabbits is severely affected by the interaction between genotype and environment [1–3]. Al-Homidan et al [4] found a decrease in the growth performance of exotic rabbit line (Spanish V-line) when compared with Saudi native rabbit breed (Jabali) raised under high environmental temperatures. Fathi et al [2] reported that the feed conversion ratio (FCR) was better for purebred rabbits (Jabali and Spanish V-line) than that of their crossbreds. The daily weight gain, FCR, litter size, and weaning survival rate were found to be the most economically productive traits negatively affected in rabbits subjected to heat stress [3].

It is well known that feed constitutes the major component of the total costs of live stock production. It accounts for 60% to 70% of the gross costs of raising rabbits for meat production [5,6]. The variable costs, including feeding, artificial insemination, replacement, and health, represent 62.1% of the total cost [3]. The financial success of any rabbit farming system depends on feed efficiency, which is usually expressed as the FCR [5,7]. In addition, improving feed efficiency in the rabbit industry reduces animal excretion and consequently reduces environmental pollution. Genetic selection for the average daily gain (ADG) under restricted feeding is one of the possible strategies to improve feed efficiency. Therefore, selection of rabbits with low residual feed intake (RFI) and high growth rate are considered the main factors affecting profitable farming [8–10]. Residual feed intake has been considered an important trait for assessing feed efficiency in livestock production for decades [7,11,12]. The difference between the actual feed intake and the expected feed intake on the basis of growth performance represents RFI in animals raised for meat production. In addition, it can be calculated for lactation and other life stages of the animal when it is not necessarily growing. Many authors report that four to six weeks is an appropriate period for the evaluation of RFI in many animal species [7,11,13,14]. Residual feed intake is calculated using multiple regression analysis. In rabbits, Drouilhet et al [15] determined the coefficients of the regression equation for the ADG and metabolic body weight in a 33-day feeding trial after weaning age. Energy requirements for maintenance would be expected to differ according to prevailing ambient temperature and genotype of the rabbits, and in turn, different results for RFI can be obtained. Rabbits under heat stress modulate physiological parameters to maintain homeothermy [16,17]. The difference in productive performance between rabbits under heat stress may be considered as a genetic basis for the selection of heat-tolerant rabbits [16].

The present study aimed to determine the relationship between RFI as a parameter of feed efficiency and growth performance, carcass characteristics, and immune response based on a feed consumption experimental period lasting six weeks, in two genotypes of growing rabbits raised under hot climatic conditions.

## MATERIALS AND METHODS

### Animals, housing, and management

This experiment was carried out at the experimental rabbit farm at the College of Agriculture and Veterinary Medicine, Qassim University, Saudi Arabia, during the hot season. The daily variation in ambient temperature (high and low) during the experimental period was recorded. The average of the maximum and minimum temperatures (mean±standard error [SE]) was 34.2°C±0.4°C and 19.1°C±0.3°C, respectively and the relative humidity ranged from 19% to 42% (SE = 0.9). Two genotypes of male rabbits aged five weeks, the Saudi local breed (Jabali; J) and an imported Spanish rabbits (V-line; V) with an average body weight of 940.0 and 916.6±15.9, respectively were used in the current study. The Spanish V-line rabbits used in this study were imported from Valencia Polytechnic University in Spain to be crossed with a population of Saudi local rabbits in 2000 [18]. The V-line was originally established from four different synthetic maternal populations in 1984, crossing crossbred males of one type with crossbred females of another type [19]. The experimental animals were assigned and confirmed to have no abnormalities and were of good health status. In total, 250 animals (125 each) were housed in individual wire mesh cages (50×40×40 cm) in a semi-closed rabbitry. The cages were equipped with feeding hoppers and drinking nipples. Feed and water were provided *ad libitum*. All animals were housed under similar housing, management, and environmental conditions. All the experimental rabbits were fed a basal diet (Table 1). All experimental procedures, animal care, and handling were performed according to the animal care instructions of scientific research deanship and approved by the committee of health research ethics and animal care (# 190208), Qassim University, Saudi Arabia.

### Growth performance and carcass analysis

Growth traits and feed intake on a dry matter basis (DMI) were individually recorded for the rabbits over 6-week experimental trail. The FCR and the ADG were calculated during the experimental period. FCR was calculated as DMI divided by body weight gain. The ADG was computed by dividing the body weight gain by 42 (the number of days of the experimental period). At the end of the experimental period, the rabbits were fasted for 12 h and then slaughtered in the morning and weighed. After bleeding, they were dissected according to Blasco and Ouhayoun [20]. The slaughtered rabbits were skinned, and hot carcasses were weighed and recorded. Organs including the liver, heart, kidney, spleen, and thymus were removed and weighed. Carcass parts (fore, mid, and hind parts) were weighed. All collected data were calculated relative to live body weight as a percentage.

### Blood collection and biochemical assay

At the end of the experiment, blood samples for biochemical analysis were collected at the time of slaughter from each rabbit into heparinized tubes. The blood samples were centrifuged (1,500×*g* for 12 min) at 4°C and the harvested plasma was stored at −20°C for further analysis. Biochemical parameters, including total protein, albumin, total cholesterol, and triglycerides, were determined using commercial kits (Biomerieux, Craponne, France). The globulin concentration was determined by subtracting the albumin concentration from the total protein concentration.

### Assay for immune response

When rabbits were ten weeks old, phytohemagglutinin P (PHA-P; Sigma Chemical Co., St Louis, MO, USA) was injected intradermally at a dose of 100 μg/kg in 0.1 mL of sterile saline into the left ear. The site of the needle was marked with permanent black ink upon injection. The ear swelling was measured before the injection and 24, 48, and 72 h afterwards using a constant tension dial micrometer (Ames, Waltham, MA, USA). The cell-mediated index was expressed as the difference between the ear thickness before and after injection. To assess the humoral immune response, total antibodies against sheep red blood cells (SRBC) were determined. Each animal was injected with 1 mL of a 10% suspension of SRBC. Sera of blood samples were collected one-week post injection to determine total immunoglobulins (Ig) using an agglutination assay. The results of antibody titers were expressed as log2 of the reciprocal of the highest dilution, giving a positive reaction. Estimation of log2 hemagglutinin titer was similar to that described by Fathi et al [21].

### Calculation of residual feed intake and statistical analysis

Expected feed intake was computed using mid-metabolic body weight (BW^0.75^) to represent maintenance requirements and the ADG to represent production requirements using multiple regression analysis. Drouilhet et al [15] suggested a formula to calculate RFI according to the following equation:


(Equation 1) $$\text{Feed intake}=243.99+1.15\times \text{ADG}+10.85\times {\left(\text{mean body weight}\right)}^{0.75}+\text{RFI}$$

In the current study, partial regression coefficients of each rabbit genotype were computed according to the following equation from the multiple regression analysis:


(Equation 2) $${OFI}_{ij}=ax{BWij}^{0.75}+bx{ADG}_{ij}+c+{EFI}_{ij}$$

Where, *OFI_ij_*, observed feed intake on a dry matter basis (g) of rabbit *i*th belonging to genotype *j*th; *EFI_ij_*, expected feed intake (g) of rabbit ith belonging to genotype *j*th; *BW_ij_*^0.75^, mean metabolic body weight of rabbit *i*th belonging to genotype *j*th (g^0.75^); *ADG_ij_*, average daily gain (g) of rabbit *i*th belonging to genotype *j*th; *a* and *b*, partial regression coefficients; *c*, intercept.

The difference between observed (OFI) and expected feed intake (EFI) for each rabbit represents the RFI. RFI was calculated for each experimental animal by using the PROC REG procedure of JMP Ver. 11, SAS Institute [22]. The results are presented as means and the pooled standard error of the mean (SEM). The mean differences between the two genotypes of all studied traits were checked using Student’s *t*-tests. The PROC CORR procedure was applied to compute the correlation coefficient between RFI and the other traits within each genotype.

## RESULTS

The differences in growth rate between the two rabbit genotypes are presented in Table 2. The rabbits of the local breed (Jabali) recorded significantly higher values for final body weight, DMI, and expected feed intake compared with those of the imported rabbits (V-line). Additionally, a significant (p<0.01) decrease (higher performance) in the FCR was found in the Jabali breed compared with the V-line breed. In terms of RFI, the Jabali breed had a negative value (−0.9 g), while the exotic breed had a positive value (+1.2 g). Accordingly, the expected feed intake for Jabali rabbits (65.3 g) was higher than that of their V-line counterparts (67.4 g).

Table 3 shows the effect of genotype on carcass traits and internal organs expressed as a percentage of live body weight. A difference (p<0.01) was detected in the dressing carcass due to genotype. The Jabali rabbits recorded the highest percentage (54.4%) compared to V-line rabbits (49.5%). Consequently, an increase (p<0.01) in the mid and hind parts was observed in the Jabali breed. No significant differences between the genotypes were detected for internal organs, including the liver, heart, and kidney. In terms of lymphoid organs, it could be noticed that the Jabali rabbits had a significantly higher percentage of spleen than that of V-line rabbits (p<0.03). The same trend (insignificant difference) was found in thymus percentage.

The biochemical blood parameters and immune response as affected by genotype of rabbit are given in Table 4. There were no significant differences between genotypes for all studied blood plasma parameters, except for total cholesterol. V-line rabbits recorded a higher cholesterol concentration (149.1 mg/dL) than Jabali rabbits (101.6 mg/dL) (p<0.01). In terms of cell-mediated immunity, it was observed that the Jabali breed exhibited a higher (0.47) cellular mediated index compared to V-line (0.44) 24 h after the PHA-P injection. The same trend was observed at 48 h and 72 h but in an insignificant manner. Antibody response to SRBC injection as a novel antigen was not significantly different between the two genotypes.

Table 5 shows the results of multiple regression analysis and the prediction equations for the expected feed consumption for each genotype of rabbit (below the table). The partial regression coefficients for daily gain had a significant effect in the computation of expected feed intake in both genotypes. Moreover, metabolic body weight had a significant (p<0.001) effect in the Jabali breed only. Generally, the intercept value did not significantly affect the computation of RFI in either the Jabali or V-line genotypes.

The phenotypic correlations between RFI and some stud ied traits are listed in Table 6. As expected, the phenotypic correlations between RFI and daily DMI or FCR were strongly positive in both genotypes. On the other hand, a weak relationship between RFI and final body weight was detected. With respect to the correlation between RFI and immune response, a low or moderate correlation was recorded in both genotypes, particularly immediately following the PHA-P injection. Residual feed consumption was negatively correlated with the relative weight of the hind part (−0.61) in the V-line. This trend almost disappeared in the Jabali breed (−0.08). A strong positive correlation was found between RFI and liver percentage, and this relationship was more pronounced in V-line rabbits. It is of interest to note that the RFI was negatively correlated with triglycerides (−0.40 vs −0.39 for Jabali and V-line, respectively), and this relationship was strong (p<0.01). Generally, an opposite relationship was observed in many traits, including the spleen, thymus, total protein, albumen, and globulin in the two rabbit genotypes.

## DISCUSSION

### Growth performance and residual feed intake

The slightly lower weaning weight observed in the V-Line rabbits could attribute to a possible differences between the two genotypes in litter size at weaning. However, this difference was more pronounced and became significant in advancing age. The results obtained for FCR fell within the normal range of most previous research studies, at 2.4 and 2.8 for Jabali and V-line, respectively. Obviously, the early fattening period showed a higher FCR than was observed during the latter period and near slaughter weight. Gidenne et al [5] recorded an FCR in the range of 2.1 to 2.3 in rabbits during the post-weaning period. In terms of RFI, the negative value recorded in the Jabali breed means a lower DMI may be expected compared to the V-line rabbits. The current results suggest that RFI is considered a good indicator of feed efficiency, but there is a disadvantage in that slow-growing animals consume a relatively small amount of feed. These animals show a more favorable RFI value [23,24]. In addition, selection for RFI results in a reduction in the FCR, accompanied by a decrease in ADG [10]. Many factors affect FCR in rabbit production. Genotype, management, feed quality, and ambient temperature are the most critical factors. When exotic breeds are transferred to another location, they may be expected to acclimatize to the new environment. Although the exotic rabbit breed (V-line) had spent a long time under the prevailing environmental temperatures in Qassim province, its performance, including ADG and FCR, was still lower than that of the local rabbits (Jabali). It seems that the V-line genotype is less adapted to ambient environmental factors. It is well documented that the imported breeds exhibit a deterioration in growth performance under high ambient temperatures compared to the local breeds. Iraqi et al [25] reported that the imported breed (V-line) showed lower body weights and weight gain than the Egyptian Gabali breed. They attributed their results to high temperatures affecting the growth rate of imported rabbits during the experimental period. Similar results were found in an experiment conducted on different genotypes by Fathi et al [2]. They stated that the imported rabbits (V-line) exhibited the lowest values of weight gain and feed intake when fed on a diet containing probiotic under summer conditions.

### Carcass analysis

The superiority observed in carcass characteristics of Jabali genotype concurs with previous studies of [2,26]. They found that the local breed and their crosses had higher carcass percentages than exotic breeds. It is well documented that there is a genetic difference between genotypes and breeds of rabbits for carcass characteristics under different environmental factors [2,7,27]. Likewise, Paci et al [28] and Belabbas et al [29] confirmed that the genetic origin significantly affects carcass traits. In an investigation to study the difference between local and foreign rabbit breeds, Abdelsalam et al [30] found a higher (p<0.03) relative percentage of spleen in the Saudi local breed compared to New Zealand White breed. They suggested that local rabbits may have a better immune response compared to their foreign counterparts under high environmental temperatures. A possible explanation for the poor performance of carcass traits associated with imported rabbit breeds could be due to the unadapted genetic potential under hot environmental conditions.

### Blood parameters and immune response

Presently, the results obtained on blood parameters determined in the two genotypes are in agreement with the previous reports conducted under the same environment. In two rabbit genotypes fed a diet supplemented with eucalyptus leaves, Fathi et al [31] reported that both genotypes (local and exotic rabbits) performed similarly for all blood biochemical parameters except for cholesterol content, where V-line had a significantly higher concentration. Concerning the immune response, it could be concluded that the Jabali breed recorded a significant improvement in cellular mediated response after 24 h of PHA-P injection. However, little literature is available concerning the effect of breed or genotype on rabbit immunity under high environmental temperatures. A slight increase in the cellular immunity of local breeds compared to exotic breeds of rabbits raised under high ambient temperature (35°C) has previously been documented [2]. A higher inflammatory response in a line of rabbits selected for higher litter size over ten generations was observed under diverse environmental challenges [32].

### Partial regression coefficients and phonotypic correlation

The importance of daily body weight gain in deriving the multiple regression equation for RFI in many species has been reported [10,11,33]. A reduction in the FCR and the ADG was found in rabbit lines selected for RFI over 10 generations [10]. In the current study, the significant positive correlations between RFI and DMI or FCR are in alignment with the finding of Drouilhet et al [15]. They found a high genetic correlation between RFI and FCR (0.96) in a rabbit line selected for RFI under an *ad libitum* feeding regimen. Similarly, Fathi et al [7] reported a strong positive correlation between RFI and both FI and FCR in two varieties of Japanese quail. In a random population of Pekin ducks, Zhang et al [8] found that the RFI was strongly correlated with FI (0.82±0.03). In addition, they recorded a positive phenotypic correlation between RFI and FCR (0.55±0.04). A negligible or low correlation was found between RFI and body weight gain (close to zero) in Japanese quail and Pekin ducks [7,11]. The phenotypic correlation between residual feed consumption and the ADG was close to zero in a commercial line of rabbits selected on post-weaning growth rate [13]. Feki et al [34] found that a negative correlated response in feed conversion was expected when selecting for daily gain. According to low correlation between RFI and immune response found in our results, selection rabbits have a lower RFI may improve immune response if the genetic correlation has the same sign. The negative relationship between RFI and the hind part of carcass is consistent with the finding of Larzul and de Rochambeau [13], who found a strong negative correlation between RFI and the hind part percentage in a commercial line of rabbits. Furthermore, Al-Saef et al [18] reported that V-line rabbits were selected for improved growth performance and carcass composition. The last findings confirmed that the V-line may possess a genetic potential beyond that of Jabali rabbits, although the local breed gains more body weight under hot environmental conditions. It is obvious that genetic structure may play an essential role in the relationship between RFI and both carcass traits and blood parameters. However, the inverse results due to genotype effect need further investigation. Using RFI instead of FCR in a selection index does not introduce new information to the index [35]. Furthermore, if RFI is directly selected without its inclusion in an index, profits would be lower, as the component traits are not weighted to obtain the maximum benefit, as the index does. Additionally, RFI in the rabbit seems to have a genetic correlation with FCR close to one, but a lower heritability [15]. Therefore, using RFI in selection would be less efficient in improving feed efficiency than directly measuring FCR [36].

## CONCLUSION

Prediction equations for the expected feed consumption for two rabbit genotypes (Jabali and V-line) raised under hot climatic conditions have been developed. The expected feed intake was significantly affected by the daily body weight gain in both genotypes, as shown in the prediction equations. Furthermore, metabolic body weight had a highly significant effect in the Jabali breed equation. Generally, Jabali rabbits exhibited good performance for growth and immune response compared to V-line rabbits. The phenotypic correlations between RFI and DMI, or FCR, were strongly positive in both genotypes. Strong negative correlation coefficients for immune response parameters were recorded in both genotypes, particularly in the period immediately after phytohemagglutinin-P injection. Considering RFI in rabbit selection programs at high temperatures is highly recommended.

## Figures and Tables

**Table 1:** Ingredients and chemical composition (as a dry matter) of the basal diet

Items	
Ingredient (%)	
Alfalfa hay	35.0
Wheat bran	8.0
Barley grain	9.0
Corn grain	20.5
Soybean meal 44% CP	22.8
Molasses	3.0
Limestone	1.2
Salt	0.25
Vitamin premix^1)^	0.27
Chemical composition (DM %)	
DE (Kcal/kg DM)^2)^	2,903
Dry matter (DM)	89.6
Crude protein	18.4
NDF	24.4
ADF	14.0
Ca	0.87
P	0.41

CP, crude protein; DM, dry matter; DE, digestible energy; NDF, neutral detergent fiber; ADF, acid detergent fiber.

1) Vitamin premix contains (per kg premix): Vit. A 4,000,000 IU, Vit. D_3_ 730,000 IU, Vit. E 3,300 mg, Vit. B_1_ 330 mg, Vit. B_2_ 1,300 mg, Vit. B_6_ 500 mg, Vit. B_12_ 305 mg, Pantothenic acid 3,500 mg, Niacin 7,000 mg, Biotin 15 mg, Folic acid 350 mg.

2) DE was calculated according to Al-Dobaib et al [24].

**Table 2:** Growth performance of two genotypes of rabbit under hot climatic conditions

Trait (g)	Genotype		SEM	p-value

	Jabali	V-Line		
IBW	940.0	916.6	15.9	0.52
FBW	2,067.9^a^	1,978.3^b^	25.81	0.05
ADG	26.9^a^	25.3^b^	0.58	<0.01
DMI	66.2	68.6	1.57	0.40
RFI	−0.9	+1.2	1.47	0.11
EFI	65.3	67.4	0.70	0.34
FCR	2.4^b^	2.8^a^	0.08	0.014

SEM, standard error of the mean; IBW, initial body weight; FBW, final body weight after 6 weeks; ADG, the average daily gain; DMI, Feed intake on a dry matter basis; RFI, residual feed intake; EFI, expected feed intake; FCR, feed conversion ratio on dry matter basis.

a,b Values within a row with different superscripts differ at p<0.05.

**Table 3:** Carcass traits and internal organs as affected by rabbit genotype

Items	Genotype		SEM	p-value

	Jabali	V-Line		
Carcass traits				
Dressing	54.4^a^	49.5^b^	0.35	<0.01
Fore part	14.8	14.1	0.13	0.06
Mid part	16.4^a^	14.4^b^	0.19	<0.01
Hind part	23.2^a^	20.9^b^	0.17	<0.01
Internal organs				
Liver	2.69	3.08	0.124	0.39
Heart	0.28	0.3	0.006	0.11
Kidney	0.61	0.64	0.013	0.33
Spleen	0.08^a^	0.06^b^	0.01	0.03
Thymus	0.18	0.17	0.007	0.72

Values expressed in g/100 slaughter weight.

SEM, standard error of the mean.

a,b Values within a row with different superscripts differ at p<0.05.

**Table 4:** Effect of rabbit genotype on the biochemical blood parameters and immune response

Trait	Genotype		SEM	p-value

	Jabali	V-Line		
Total protein (g/dL)	6.09	5.95	0.11	0.46
Albumin (g/dL)	3.91	3.79	0.042	0.26
Globulin (g/dL)	2.18	2.17	0.067	0.93
Total cholesterol (mg/dL)	101.6^b^	149.1^a^	6.3	<0.01
Triglycerides (mg/dL)	133.3	117.8	6.87	0.28
CMI (h)				
24	0.47^a^	0.44^b^	0.01	0.05
48	0.31	0.28	0.02	0.2
72	0.18	0.17	0.01	0.35
AT	5.8	5.5	0.16	0.41

SEM, standard error of the mean; CMI, cellular mediated index; AT, antibody titer against sheep red blood cells.

a,b Values within a row with different superscripts differ at p<0.05.

**Table 5:** Partial regression coefficients for factors affecting expected feed intake of two rabbit genotypes

Parameter estimate	Partial regression coefficient		Prob.	
	
	Jabali	V-Line	Jabali	V-Line
Intercept	1.87	7.27	0.90	0.69
ADG (g)	0.48*	0.99*	0.04	0.03
(BW)^0.75^ (g)	0.21**	0.15	<0.001	0.13

n = 125 individual records/genotype,

* p<0.05,

** p<0.01.

Prediction equations: Y = 0.48ADG+0.21(BW)^0.75^+1.87(Jabali breed), Y = 0.99ADG+0.15(BW)^0.75^+7.27(V-Line), where: Y stands for expected feed intake; ADG, average daily gain; (BW)^0.75^, metabolic body weight.

**Table 6:** Phenotypic correlation between residual feed intake and some studied traits in two genotypes of rabbit

Trait	Phenotypic correlation	

	Jabali	V-Line
DMI	0.93**	0.90**
FCR	0.76**	0.79**
FBW	0.07	0.08
CMI (h)		
24	−0.25*	−0.29*
48	−0.18	−0.30*
72	−0.13	−0.19
AT	−0.21	0.05
Dressing carcass	0.20	−0.45**
Forepart	0.03	0.07
Mid-part	0.30*	−0.18
Hind-part	−0.08	−0.61**
Liver	0.41**	0.77**
Heart	−0.07	0.35*
Spleen	0.20	−0.65**
Thymus	0.09	−0.20
Total protein	0.08	−0.15
Albumin	−0.53*	0.15
Globulin	0.42*	−0.26*
Cholesterol	0.25	−0.20
Triglycerides	−0.40**	−0.39**

DMI, feed intake on a dry matter basis; FCR, feed conversion ratio; FBW, final body weight; CMI, cellular mediated index; AT, antibody titer against sheep red blood cells.

* p<0.05,

** p<0.01.

## References

[1] Ragab M, Elkhaiat I, Younis H, Ahmed M, Helal M (2022). Genotype by heat conditions interaction effects on growth and litter traits in rabbits. Front Vet Sci.

[2] Fathi M, Abdelsalam M, Al-homidan I, Ebeid T, El-Zarei M, Abou-Emera OK (2017). Effect of probiotic supplementation and genotype on growth performance, carcass traits, hematological parameters and immunity of growing rabbits under hot environmental conditions. Anim Sci J.

[3] Cartuche L, Pascual M, Gómez EA, Blasco A (2014). Economic weights in rabbit meat production. World Rabbit Sci.

[4] Al-Homidan I, Fathi M, Abdelsalam M (2022). Effect of propolis supplementation and breed on growth performance, immunity, blood parameters and cecal microbiota in growing rabbits. Anim Biosci.

[5] Gidenne T, Garreau H, Drouilhet L, Aubert C, Maertens L (2017). Improving feed efficiency in rabbit production, a review on nutritional, technico-economical, genetic and environmental aspects. Anim Feed Sci Technol.

[6] Adanguidi J (2020). Analysis of the profitability and competitiveness of rabbit value chains in benin. J Agric Sci.

[7] Fathi MM, Al-Homidan I, Ebeid TA, Galal A, Abou-Emera OK (2019). Assessment of residual feed intake and its relevant measurements in two varieties of Japanese quails (Coturnixcoturnix japonica) under high environmental temperature. Animals.

[8] Drouilhet L, Gilbert H, Balmisse E (2013). Genetic parameters for two selection criteria for feed efficiency in rabbits. J Anim Sci.

[9] Drouilhet L, Achard CS, Zemb O (2015). Direct and correlated responses to selection in two lines of rabbits selected for feed efficiency under ad libitum and restricted feeding: I. Production traits and gut microbiota characteristics. J Anim Sci.

[10] Garreau H, Ruesche J, Gilbert H (2019). Estimating direct genetic and maternal effects affecting rabbit growth and feed efficiency with a factorial design. J Anim Breed Genet.

[11] Zhang Y, Guo ZB, Zhang Z, Xie M, Hou S (2017). Genetic parameters for residual feed intake in a random population of Pekin duck. Asian-Australas J Anim Sci.

[12] Jin S, Xu Y, Zang H (2020). Expression of genes related to lipid transport in meat-type ducks divergent for low or high residual feed intake. Asian-Australas J Anim Sci.

[13] Larzul C, de Rochambeau H (2005). Selection for residual feed consumption in the rabbit. Livest Prod Sci.

[14] Yuan J, Wang K, Yi G (2015). Genome-wide association studies for feed intake and efficiency in two laying periods of chickens. Genet Sel Evol.

[15] Drouilhet L, Garreau H, Tudela F Genetic determinism of feed efficiency in rabbit: Analysis of a selection experiment for two criteria of feed efficiency. https://hal.archives-ouvertes.fr/hal-01190213.

[16] Jimoh OA, Ewuola EO (2018). Thermophysiological traits in four exotic breeds of rabbit at least temperature-humidity index in humid tropics. J Basic Appl Zool.

[17] Ferraz PFP, Hernández-Julio YF, Ferraz GAS (2019). Decision trees for predicting the physiological responses of rabbits. Animals.

[18] Al-Saef AM, Khalil MH, Al-Dobaib SN, Al-Homidan AH, García ML, Baselga M (2008). Comparing Saudi synthetic lines of rabbits with the founder breeds for carcass, lean composition and meat quality traits. Livest Res Rural Dev.

[19] Mínguez C, Sanchez JP, El-Nagar AG, Ragab M, Baselga M (2016). Growth traits of four maternal lines of rabbits founded on different criteria: comparisons at foundation and at last periods after selection. J Anim Breed Genet.

[20] Blasco A, Ouhayoun J (1993). Harmonization of criteria and terminology in rabbit meat research. Revised Proposal. World Rabbit Sci.

[21] Fathi MM, Ali RA, Qureshi MA (2003). Comparison of immune responses of inducible nitric oxide synthase (iNOS) hyper- and hypo responsive genotypes of chickens. Int J Poult Sci.

[22] SAS Institute (2013). JMP Version 11 User’s guide.

[23] Berry DP, Crowley JJ (2012). Residual intake and body weight gain: a new measure of efficiency in growing cattle. J Anim Sci.

[24] Willems OW, Miller SP, Wood BJ (2013). Assessment of residual body weight gain and residual intake and body weight gain as feed efficiency traits in the turkey (Meleagris gallopavo). Genet Sel Evol.

[25] Iraqi MM, Afifi EA, Baselga M, Khalil MH, Garcia ML Additive and heterotic components for post weaning growth traits in a crossing project of V-line with Gabali rabbits in Egypt.

[26] Al-Dobaib SN, Khalil MH, Hashad M, Al-Saef AM (2007). Growth, carcass and caecal traits in V-line and crossbred rabbits fed diet containing discarded dates. World Rabbit Sci.

[27] Wang J, Su Y, Elzo MA, Jia X, Chen S, Lai S (2016). Comparison of carcass and meat quality traits among three rabbit breeds. Korean J Food Sci Anim Resour.

[28] Paci G, Cecchi F, Preziuso G, Ciampolini R, D’Agata M (2012). Carcass traits and meat quality of two different rabbit genotypes. Ital J Anim Sci.

[29] Belabbas R, de la Luz García ML, Ainbaziz H (2019). Growth performances, carcass traits, meat quality, and blood metabolic parameters in rabbits of local Algerian population and synthetic line. Vet World.

[30] Abdelsalam M, Al-Homidan I, Ebeid T (2019). Effect of silver nanoparticle administration on productive performance, blood parameters, antioxidative status, and silver residues in growing rabbits under hot climate. Animals.

[31] Fathi MM, Abdelsalam M, Al-Homidan I (2019). Supplemental effects of eucalyptus (Eucalyptus camaldulensis) leaves on growth performance, carcass characteristics, blood biochemistry and immune response of growing rabbits. Ann Anim Sci.

[32] Argente MJ, García ML, Zbyňovská K, Petruška P, Capcarová M, Blasco A (2019). Correlated response to selection for litter size environmental variability in rabbits’ resilience. Animal.

[33] Badawe MI, Abd El-Hameid EF, Fathi MM, Zein El-Dein A (2005). Prediction of feed intake and residual feed consumption in laying hen chickens. Egypt Poult Sci.

[34] Feki S, Baselg M, Blas E, Cervera C, Gómez EA (1996). Comparison of growth and feed efficiency among rabbit lines selected for different objectives. Livest Prod Sci.

[35] Kennedy BW, Van der Werf JH, Meuwissen TH (1993). Genetic and statistical properties of residual feed intake. J Anim Sci.

[36] Blasco A, Nagy I, Hernández P (2018). Genetics of growth, carcass and meat quality in rabbits. Meat Sci.

